# An experimental study of liquid micro-jets produced with a gas dynamic virtual nozzle under the influence of an electric field

**DOI:** 10.3389/fmolb.2023.1006733

**Published:** 2023-01-19

**Authors:** Bor Zupan, Gisel Esperanza Peña-Murillo, Rizwan Zahoor, Jurij Gregorc, Božidar Šarler, Juraj Knoška, Alfonso M. Gañán-Calvo, Henry N. Chapman, Saša Bajt

**Affiliations:** ^1^ Laboratory for Fluid Dynamics and Thermodynamics, Faculty of Mechanical Engineering, University of Ljubljana, Ljubljana, Slovenia; ^2^ Center for Free-Electron Laser Science CFEL, Deutsches Elektronen-Synchrotron DESY, Hamburg, Germany; ^3^ Laboratory for Simulation of Materials and Processes, Institute of Metals and Technology, Ljubljana, Slovenia; ^4^ Departamento de Ingeniería Aeroespacial y Mecánica de Fluidos, Universidad de Sevilla, Sevilla, Spain; ^5^ Laboratory of Engineering for Energy and Environmental Sustainability, Universidad de Sevilla, Sevilla, Spain; ^6^ The Hamburg Centre for Ultrafast Imaging, Hamburg, Germany; ^7^ Department of Physics, Universität Hamburg, Hamburg, Germany

**Keywords:** gas dynamic virtual nozzle, electric field, micro-jet, experimental study, jetting modes, flow-focusing, Taylor cone

## Abstract

The results of an experimental study of micro-jets produced with a gas dynamic virtual nozzle (GDVN) under the influence of an electric field are provided and discussed for the first time. The experimental study is performed with a 50% volume mixture of water and ethanol, and nitrogen focusing gas. The liquid sample and gas Reynolds numbers range from 0.09–5.4 and 0–190, respectively. The external electrode was positioned 400–500 μm downstream of the nozzle tip and an effect of electric potential between the electrode and the sample liquid from 0–7 kV was investigated. The jetting parametric space is examined as a function of operating gas and liquid flow rates, outlet chamber pressure, and an external electric field. The experimentally observed jet diameter, length and velocity ranged from 1–25 μm, 50–500 μm and 0.5–10 m/s, respectively. The jetting shape snapshots were processed automatically using purposely developed computer vision software. The velocity of the jet was calculated from the measured jet diameter and the sample flow rate. It is found that micro-jets accelerate in the direction of the applied electric field in the downstream direction at a constant acceleration as opposed to the standard GDVNs. New jetting modes were observed, where either the focusing gas or the electric forces dominate, encouraging further theoretical and numerical studies towards optimized system design. The study shows the potential to unlock a new generation of low background sample delivery for serial diffraction measurements of weakly scattering objects.

## 1 Introduction

Serial femtosecond crystallography (SFX) (Chapman et al., 2011) utilizes femtosecond X-ray pulses generated by an X-ray Free-Electron Laser (XFEL) to produce diffraction patterns of crystals and provide insight into their internal molecular structure. In a particular scheme of SFX (Stagno et al., 2017; Gisriel et al., 2019; Williamson et al., 2022), protein microcrystals are transferred to the X-ray beam in a liquid suspension focused into a thin micro-jet. The transfer is achieved using gas dynamic virtual nozzles (GDVNs) (Gañán-Calvo, 1998; DePonte et al., 2008), which employ forces exerted by a co-flowing and expanding gas that creates a virtual nozzle, accelerating the liquid and focusing the diameter of the jet. GDVNs are now typically 3D printed using a two-photon polymerization (2pp) process, which enabled reproducible manufacturing of micro-nozzles with complex geometries and with submicron precision (Nelson et al., 2016; Knoška et al., 2020).

Application-driven requirements of the micro-jet characteristics require continued investigations for improving the design and operation of GVDNs. The introduction of the European XFEL facility with megahertz pulse frequency requires very fast (∼100 m/s) and thin jets (∼1 μm) with a length of at least 50 μm. Such technical specifications for the jet pose additional challenges and stimulate a search for unconventional solutions. The pioneering theoretical and experimental development in the field of electrohydrodynamics, in particular electrosprays (Gañán-Calvo and Montanero, 2009; Gañán-Calvo et al., 2018), have opened new possibilities in the field of flow focusing. By applying an electrical potential between the liquid and the external electrode, an additional force is added to the axis of the micro-jet. Electric charge accumulates at the liquid-gas interface, thus accelerating the jet due to the presence of an electric force. A hybrid operational approach of focusing and accelerating jets with gas-focusing and an electric field can provide an alternative to impractical miniaturization for achieving the objective of required stable, thin, long and fast jets. With this novel alternative, the jets could be produced with necessary characteristics without losing system robustness. A theoretical study of aerodynamically stabilized Taylor cone (TC) jets (Cruz-Mazo et al., 2019) reports a possible 50% increase in maximum jet velocity for both pure electrospray and flow focusing, motivating additional research on this topic.

This paper reports the first experimental study of gas flow rate, sample liquid flow rate and electric potential influence on jet parameters such as length, diameter, and velocity of micro-jets produced with GDVN under the influence of an electric field. New modes of jet operation different from the classical GDVN flow are also observed and discussed. The present findings are of interest for future SFX experiments of weakly scattering and other objects where very thin jets are required to reduce background scattering.

## 2 Materials and methods

### 2.1 Experimental setup

**FIGURE 1:**
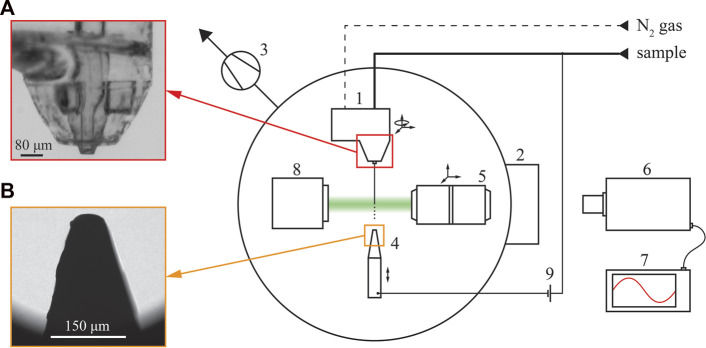
Schematic of the experimental setup for flow-focused micro-jets in the electric field located at CFEL, Hamburg. An optical image detail **(A)** provides a close-up of the GDVN while **(B)** shows the exposed electrode tip. Ice can be seen forming on the electrode as the irregular contour on the left.

The experimental setup was designed and built at the Center for Free-Electron Laser Science (CFEL) in Hamburg, Germany. All experiments reported here were performed with this setup. A schematic of the setup design is shown in Figure 1. The numbers in the brackets in this section refer to the labelled items in Figure 1. The GDVN (1) was located in a vacuum chamber (2) and was mounted on a piezoelectric actuated stage that allows four degrees of freedom: three degrees of translation and one degree of rotation. Turbopumps (3) are used to evacuate the chamber when measurements of the jet in a vacuum environment are performed. The electrode (4) distance to the nozzle is controlled with one piezo stage. Piezo stages are also used to position the plan apochromatic objective ELWD 20x/0.42 (5) in three orthogonal directions.

The images of jets were captured by a high-resolution incoherent imaging system described in Knoška et al. (2020). The system consists of a Zyla 5.5 sCMOS camera (6) with the ability to record 100 frames per second (FPS) at 5.5 megapixels. With the 20x objective, spatial measurements of the jets were made with a resolution of 0.31 μm. The camera was triggered by an external digital delay generator DG635 (7), which was synchronized with the illumination. The illumination was provided by laser-induced fluorescence (iLIF) using a Quantel double-pulse Nd-YAG laser with the primary wavelength in the visible range at 532 nm. However, the laser was only used to produce a single pulse for each exposure. The emitted light was absorbed by a rhodamine suspension which emitted in the visible spectrum with a wavelength of 568 nm. An optical collimator/coupler was used to capture the bright field illumination from the rhodamine, which was transferred into the vacuum chamber *via* an optical fiber. The light was reflected from an optical mirror (8) and provided spatially-incoherent short-pulse illumination for the experiment in the vacuum chamber.

The sample liquid, a mixture of 50% vol EtOH and H_2_O, was pumped with two high-precision Nemesys syringe pumps. Nitrogen gas at high pressure was used for focusing. The high pressure was reduced with a digital pressure regulator, and the mass flow rate was measured with Bronkhorst (F-111B-200 and 500) gas flow meters. The dotted and bold lines in Figure 1 indicate the gas and sample conduits.

A high-voltage power supply (FuG HCP 35-20000) generated the electric potential between the electrode and the liquid jet (9). At 30 W and 1.5 mA, the power supply can deliver voltages up to 20 kV. A single voltage polarity was used in the present study: the positive electrode was submerged in the sample liquid, and the negative electrode was located downstream from the nozzle. This exposed electrode was a tungsten rod with a diameter of 200 µm with a tip formed into a cone of 25° vertex angle. The tip of the rod was rounded to a radius of 27.5 µm.

### 2.2 Nozzle design and non-dimensional parameters

**FIGURE 2:**
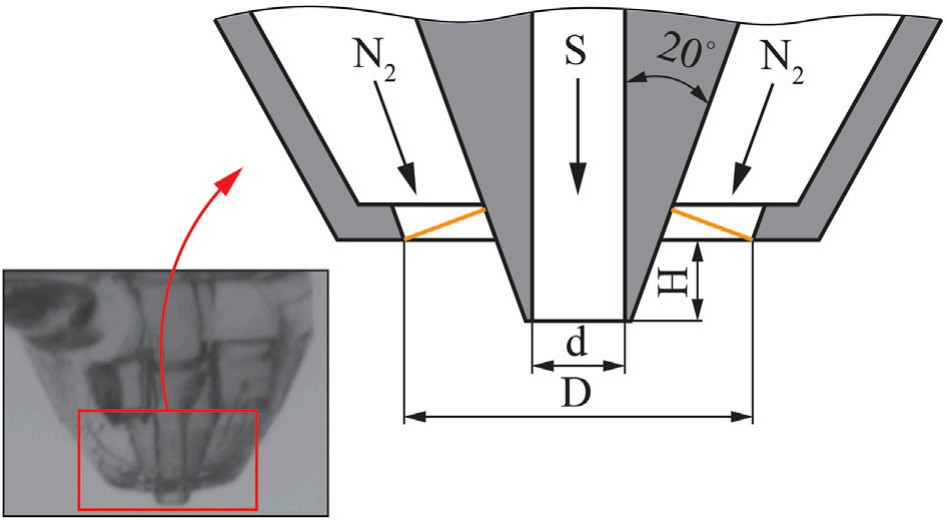
Design of the GDVN for flow focusing in an electric field. D = 150 μm, d = 40 μm and H = 35 μm. The orange lines show the cross section of the area that gas flows, used to define the Reynolds number.

The GDVNs were printed using a Nanoscribe Photonic Professional GT two-photon polymerization printer using IP-S resin deposited onto an ITO coated glass slide with 0.5-micron precision. A detailed description of the nozzle fabrication process can be found in (Knoška et al., 2020) under the Device design and fabrication section. The main geometrical parameters of the nozzle are shown in Figure 2. The nozzle exhibits a characteristic protruding inner capillary, carrying the sample liquid (denoted by S). This design avoids any accumulation of charged liquid at the outer (gas) funnel (denoted by N_2_), which would otherwise disrupt the jetting.

The sample and gas flow rates are expressed in a non-dimensional form through the Reynolds number. The gas Reynolds number is defined for a conical annulus with outer diameter *D* and surface normal parallel to the jet velocity vector (the cross-section of this annulus is shown as the orange lines in Figure 2). The sample and gas Reynolds numbers, Re_*s*_ and Re_*g*_, are given by:

$$Re_{s}=\frac{\rho_{s}v_{s}d}{\mu_{s}}=\frac{4{\dot{m}}_{s}}{\pi d\mu_{s}}$$

$$Re_{g}=\frac{\rho_{g}v_{g}D_{h}}{\mu_{g}}=\frac{4{\dot{m}}_{g}}{\mu_{g}P}$$

with density ρ , velocity v , dynamic viscosity μ , mass flow rate $\dot{m}$ , hydraulic diameter $D_{h}$ , wetted perimeter P , and indices g and s denoting the gas and sample, respectively. In addition, the non-dimensional jet length L is expressed as the ratio between the length of the jet l and the distance between the outer electrode and the tip of the nozzle $l_{0}$ , which is usually between 400 and 500 μm:

$$L=\frac{l}{l_{0}}$$

The ranges of experimentally observed dimensionless numbers and electric potential are given in Table 1.

**TABLE 1:** Ranges of dimensionless numbers and electric potential.


$Re_{s}$	0.09−5.4
$Re_{g}$	0−190
L	0.16−1
$EP\left[kV\right]$	0−7

### 2.3 Automated jet-shape-processing method

The miniature size of the experiment represents the main difficulty and limitation for measurements, such as accurate spatial and temporal *in-situ* measurements of velocity, pressure, and temperature. The chaotic nature of jet perturbations imposes alterations of the jet structure on multiple temporal scales. The length of the jet constantly changes, with length fluctuations as large as 20% (Gañán-Calvo et al., 2019). Thus, measurements over multiple frames are required to obtain the average micro-jet shape, resulting in hundreds of individual frames for each case. The measurement of particular parameters and profiles of jets in each individual frame by hand is time-consuming with questionable precision. Therefore, post-processing Python-based software for jet characterization was developed for an automated assessment of jet shape in every frame. The library utilizes Numpy (Harris et al., 2020), Scikit (Pedregosa et al., 2011) and Scipy (Virtanen et al., 2020).

**FIGURE 3:**
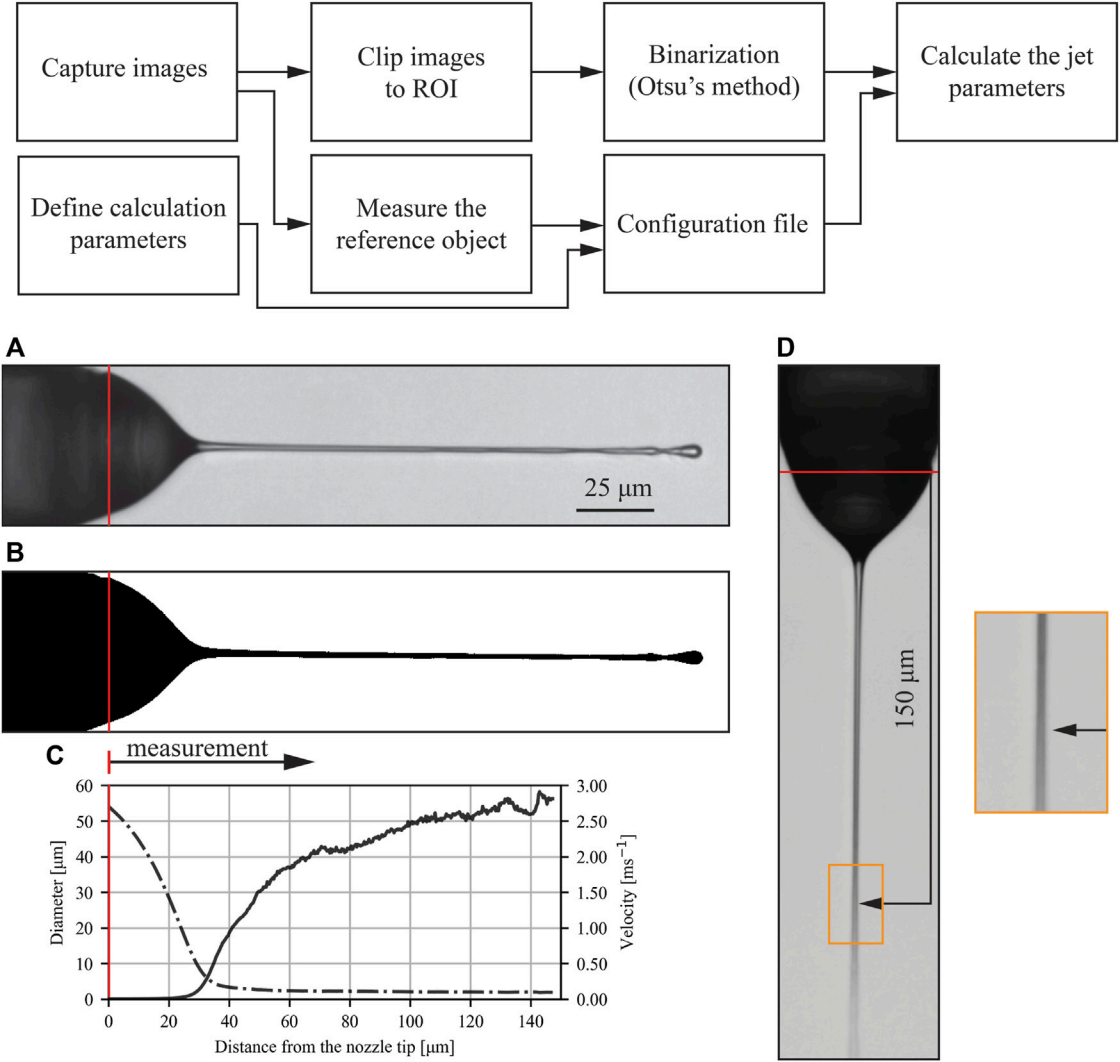
Automated jet-shape-processing method pipeline is shown on the top of the image. Detail **(A)** shows the raw image of the jet and its binarized form in detail **(B)**. The result from the post processing is shown in detail **(C)**, where the dotted line represents the diameter measurement and the full line represents the calculated velocity. The averaged image of the whole stack is shown in detail **(D)**. The location of the average breakup is enlarged on the right. The red line marks the nozzle tip location all details. The following parameters are used for this case: GFR = 0.1 mg/min, SFR = 0.5 μl/min, EP = 5 kV, L = 0.405.

**FIGURE 4:**
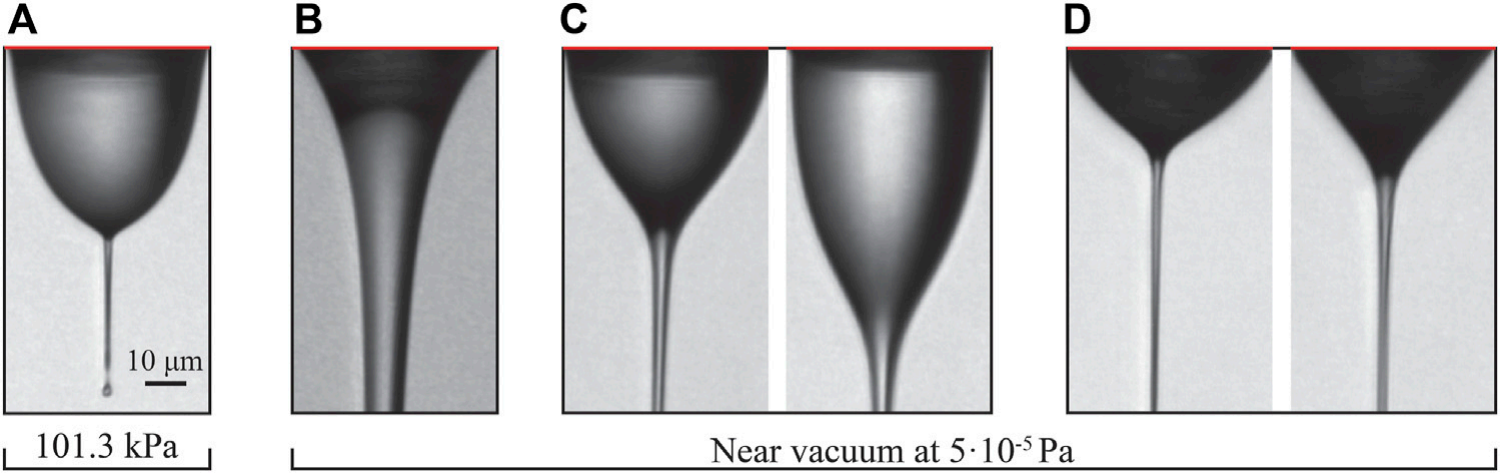
The shape of the Taylor cone as a function of GFR, SFR and EP. The following parameters were used: **(A)** GFR = 9.1 mg/min, SFR = 0.25 μl/min, EP = 7 kV, **(B)** GFR = 41.3 mg/min, SFR = 12 μl/min, EP = 0 kV, left **(C)** GFR = 15 mg/min, SFR = 1 μl/min, EP = 6 kV, right **(C)** GFR = 15 mg/min, SFR = 2.5 μl/min, EP = 6 kV, left **(D)** GFR = 19.2 mg/min, SFR = 0.5 μl/min, EP = 5 kV, right **(D)** GFR = 18.9 mg/min, SFR = 1 μl/min, EP = 6 kV.

The pipeline of the measurement method is shown in Figure 3. Images are captured in a TIFF stack format and are clipped to the region of interest (ROI) for easier manipulation. An example is shown in detail in Figure 3A. The stack is then fed into a binarization algorithm, which is implemented using Otsu’s method (Otsu, 1979), shown in detail in Figure 3B. Together with the binarized stack some additional parameters are needed and are specified in the configuration file. The pixel width is measured in the first frame of the stack from a reference object. The starting location of the jet calculation can be either specified (such as the nozzle tip) or calculated (meniscus-to-jet transition). The meniscus-to-jet transition is defined as the point along the jet axis where the standard deviation of the jet diameter in the next ten pixels along the jet axis in the direction of flow is below 1. This transition criterion was experimentally set by comparing the results over different jetting regimes (excluding b in Figure 4, where there is no clear meniscus). With all required data fed into the algorithm, the complete geometrical parameters of the jet were calculated for every frame. The set of geometrical parameters consisted of the jet start and breakup point, its diameter along the jet axis and the length of the jet.

Measurements of the jet diameter were averaged at each distance from the nozzle tip over all acquired images. Because the length of the jet changes from image to image, there are only a few rare observations of the longest jets. Therefore, the diameter was evaluated only up to the distance where at least 90% of the images contained the jet. This average measured diameter over the length of the stable jet is shown in Figure 3C with the dotted line. A simple jet velocity calculation resulting from the conservation of mass for the liquid phase and jet diameter is shown in Figure 3C with the full line. All presented results were calculated with 100 captured frames, acquired at 14 frames per second and with an exposure time of 150 μs each. The length of the jet, used in the calculation of *L*, is the average value of the jet length of those 100 captured frames.

Our image-based approach to the measurement of jet parameters is similar to the method described by Patel et al. (2022) whereby jets were automatically brought to a fixed position. This image-based method contrasts with the method developed by Nazari et al. (2020), where the jet parameters are calculated from Particle Tracking Velocimetry (PTV) of droplets. The advantage of the jet-shape-processing method over the PTV is the ability to obtain the velocity distribution along the whole longitudinal axis of the jet, as opposed to the region with droplets occurrence. Another method capable of producing such results is by taking a double exposure image of jets containing tracer particles, using pulse spacings shorter than 1 µs (Knoška et al., 2020). Dual-pulse imaging achieves a more accurate measurement of the velocity than the jet-shape-processing method, while using the same iLIF illumination and optical systems. This is because the velocities in particle tracking are deduced from spatial measurements made over larger distances than the width of the jet itself. However, jet-shape-processing gives an unblurred image of the jet structure.

To justify the use of the averaging over a series of frames at specific locations, the overall stability of the jet is shown in Figure 3D. Here, an average intensity image of 100 frames is displayed. The location where the jet breaks into droplets can be easily spotted as the average image becomes blurred and the jet trace widens. For Figure 3D, this location is at approximately 150 μm downstream of the tip as determined by visual inspection. This is consistent with the results (Figure 3C) of the 90% criteria described above.

Including the automatically measured variables, we collected the sample flow rate (SFR), gas flow rate (GFR), electric potential (EP), vacuum pressure and the complete jet shape for every frame and calculated the velocity as a function of the jet diameter $d_{j}$ . The velocity of the jet $v_{j}$ is calculated as

$$v_{j}\left(d_{j}\right)=\frac{4\;SFR}{\pi d_{j}^{2}}$$

The equation above gives a first approximation of the jet velocity, where no velocity gradient in the radial direction of the jet is considered. Based on the result from Zahoor et al. (2021a), we can assume that the largest error of the velocity calculation occurs in the meniscus or Taylor cone, where the largest velocity gradient in the radial direction is present. In the jet region, where the velocity gradient is negligible, this approximate method should provide pixel-accurate velocity measurements. Based on this, all velocity measurements in Figures 7–9 were calculated from the meniscus-to-jet transition onwards.

### 2.4 Measurement uncertainty

Major measurement uncertainty originates in the jet-shape-processing method, where the binarization process fixes the resolution to pixel accuracy. In the jet’s longitudinal axis, the pixel accuracy induces negligible error due to a large number of pixels. The error grows substantially in the radial direction, where the number of pixels across the jet varies from around 4 for thinnest jets to more than 70 for the thickest jets. This places the diameter measurement uncertainty between 2.9% and 50% at the extremes and about 30% for the jets with diameters just below 2 µm. The radial measurement error is partially reduced by averaging over multiple frames. Given the measurement uncertainty and considering the averaging effect, the results are used to represent trends and do not give values to sub-micron precision.

Gas flow rate meters operate with an uncertainty of 0.1% full scale, which amounts to 0.375–0.94 mg/min or 2.5%–13%. The syringe pump can achieve sample flow rates below 1 nl/min. For the flow range used in the present study, the measurement error of the sample flow was negligible. Voltage measurement uncertainty was 0.1%.

### 2.5 Selection of fluids

Nitrogen gas has excellent focusing properties in combination with high voltage fields as it is inert, dielectric and non-electron attaching. Its breakdown voltage is 1.15 times that of the air or roughly 35 kV/cm at 1 atm. Because of enhanced electrical properties, nitrogen was selected as the focusing gas instead of helium, typically used for SFX.

A combination of water and ethanol was used based on the well-known properties of both liquids. In the current experimental design, some form of an alcohol solution is necessary to counter the freezing of water. Although not experimentally measured, some degree of molecular dissociation of the ethanol-water solution is expected, changing the electric properties, such as the conductivity of the mixture in the course of the experiment.

## 3 Results

The observed diversity of different jetting modes and Taylor cone shapes is astounding. The most typical of them are shown in Figure 4. There seems to be fierce competition between the forces resulting from the expanding gas and the electric field, giving rise to different meniscus (or Taylor cone) shapes and different jetting regimes.

### 3.1 Taylor cone variation and jetting modes

Under atmospheric pressure conditions, only one stable jetting mode is observed (Figure 4A). Here, GFR has little to no effect on the shape of the Taylor cone. A characteristic thin and short jet is formed at the tip of the cone, which protrudes from the tip at a distance similar to that of the diameter of the inner capillary. Droplets of constant diameter are produced at the breakup point, which is fixed and does not change. This regime is usually referred to as “tip streaming” (Montanero and Ganán-Calvo, 2021) as opposed to “dripping” or “jetting,” which implies large oscillations of the meniscus at the capillary source or a long capillary jet breaking up with certain irregularity, respectively. The breakup mechanism (Rayleigh-Plateau instability, or varicose breakup) is the same as in the other jets with applied EP, described later.

In a vacuum, three characteristic Taylor cone shapes are observed. Figure 4B shows the typical meniscus shape when no electric potential is applied and/or at high sample flow rate. The liquid-gas interface assumes a continuously tapering convex curve leading to the end of the jet (end not shown in Figure 4B), where a breakup mechanism similar to that of regular jets in absence of EP is observed (Zahoor et al., 2021b). The electric potential at high sample flow rate has a negligible effect on the meniscus. At applied voltages below 5-6 kV (Figure 4C) the Taylor cone protrudes again. Unlike the atmospheric pressure situation, the surface of the Taylor cone takes on a more continuous shape as it approaches the jet. A thicker jet is formed (left image) that is stable only at certain gas and sample flow rates, outside of which a whipping regime and a cone-pulsating regime occur. In the latter, the Taylor cone pulsates in size and in some cases, may even detach, and a large droplet travels downstream, still attached to the jet. At a higher sample flow rate, above 1.5 μl/min (right image in Figure 4C), the Taylor cone protrudes even further, and the jet becomes highly unstable. By setting the sample flow rate even higher, the jetting transitions completely to the cone-pulsating regime. In some cases, even a bridging regime can occur, where the jet reaches the exposed electrode and no breakup occurs. Figure 4D shows the most common mode, stable over a wide range of gas and sample flow rates, and electric potentials. Here, the Taylor cone geometry change is minor and observed mainly as a function of sample flow rate and electric potential.

**FIGURE 5:**
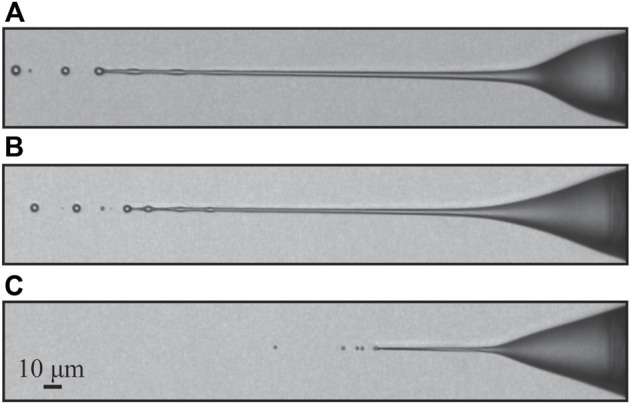
**(A)** Strong jetting mode (L = 0.73) and **(B–C)** transitional modes (L = 0.32 for case **(C)**) in atmospheric pressure captured for the same test case: GFR = 52.9 mg/min, SFR = 0.75 μl/min, EP = 6.1 kV.

Two distinctive jetting modes are observed in a vacuum and at atmospheric pressure. The first mode (Figure 4A), which we call a “weak jetting mode” or “tip streaming mode”, is stable under both pressure conditions (vacuum and in the air), while the second mode (Figure 5A), denoted a “strong jetting mode”, only persists as a stable jet in vacuum. Under unstable jetting conditions, it is likely that both modes alternate between intermediate transition modes. Such transitions are displayed in Figures 5B, C.

An additional jetting mode can be achieved if the electrode is placed close enough to the nozzle tip, the sample flow rate is above 1 μl/min, the gas flow rate is close to zero, and the applied voltage is above 6 kV: the bridging regime. Here the jet reaches the outer electrode, where it is discharged. Due to little or no stabilizing effect of the focusing gas, a pulsing of the Taylor cone is often observed. At lower than optimal sample flow rates, the jet breaks right before the outer electrode. At higher than optimal sample flow rates, a cone-pulsating regime occurs.

### 3.2 Breakup and droplets

Growing perturbations induce the well-documented Rayleigh-Plateau instability (Rayleigh, 1878) (Figures 5A, B). The perturbation develops as droplets larger than the jet diameter, when the flow is properly focused. These then break off as a singular entity, sometimes trailing a transient liquid ligament. This ligament either recoils in the droplet or breaks off into satellite droplets. This mechanism can be observed in experiments and related numerical simulations of GDVNs (Zahoor et al., 2018c).

**FIGURE 6:**
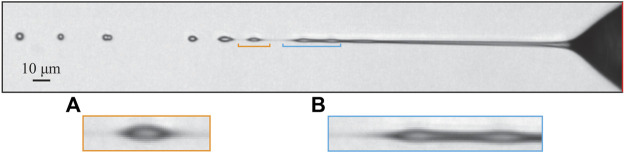
Most common geometry of the breakup. Detail **(A)** shows the elongated droplet with thin, almost invisible jet connecting the droplet to the jet structure. In detail **(B)** elongated perturbations are displayed together with an extended thin liquid ligament brought about by the presence of electric charges (Rubio et al., 2021). The image was produced using the following parameters: GFR = 24.7 mg/min, SFR = 1 μl/min, EP = 6 kV, L = 0.47.

The presence of the electric potential affects the breakup mechanism in the following way. The perturbations rarely grow to large diameters but are almost always elongated along the jet (Figure 6b). This is due to the additional stabilizing effect of the axial electric field as opposed to that of surface tension. The jet tip thus transforms into smaller, elongated droplets connected by very thin, submicron jets (Figure 6a). It is still unclear whether these narrow connecting jets are part of the former jet or were created due to Rayleigh jetting (Duft et al., 2003). Still, the most probable scenario is that they result due to their delayed recoil associated with the presence of charges (Rubio et al., 2021, see their Figures 8–10, 12). To be able to answer this question, the jet breakup has to be captured with a higher frame rate.

### 3.3 Effect of flow rates and applied voltage on diameter and velocity profiles

**FIGURE 7:**
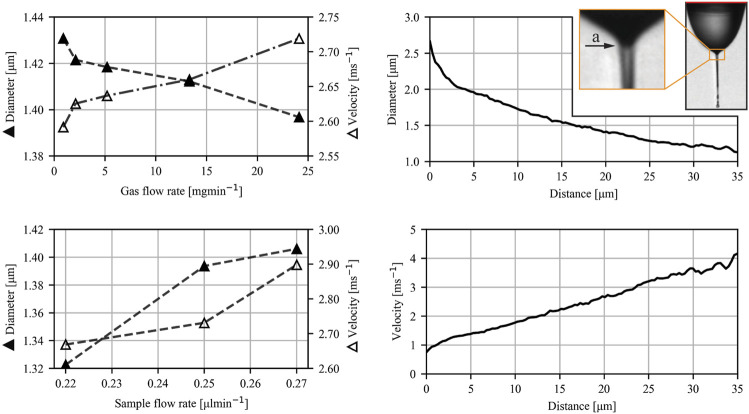
Diameter and velocity profiles for atmospheric pressure conditions. Figures on the left show the influence of the gas and sample flow rates on jet diameter and velocity 20 μm downstream of the meniscus-to-jet transition (denoted by an arrow (a) shown in the insert on the right). The figures on the right represent the typical diameter (solid triangle) and velocity profile (open triangle) as a function of distance from the meniscus-to-jet transition of a jet under atmospheric pressure. EP = 7 kV for all cases.

Figure 7 shows the typical velocity and diameter profiles, and the influence of the gas and sample flow rates on the jet diameter and velocity under atmospheric pressure conditions. Due to the nozzle design, only a very narrow range of applied voltages, about 7 kV, leads to stable jetting in the atmosphere. The protruding nozzle limits the exposure time of the liquid-gas interface to the expanding gas, leading to lower kinetic energy transmission, which, combined with a lower expansion ratio of the gas (into the ambient atmosphere), provides a lower stabilizing and flow-focusing effect. All figures were therefore obtained for cases with EP = 7 kV while GFR = 13.3 mg/min and SFR = 0.25 μl/min were used for the velocity and diameter measurements shown in Figure 7. The influence of the gas flow rate on the jet was measured at constant SFR = 0.25 μl/min, and the influence of sample flow rate on the jet was measured at constant GFR = 9.1 μl/min. The dimensionless length L remained constant (0.51). The distance along the jet was determined relative to the meniscus-to-jet transition denoted by the arrow (a) in Figure 7. The zero position was determined automatically by measuring the standard deviation of ten consecutive diameter positions in a single image over the whole jet. The threshold value for the standard deviation was experimentally set to 1. We assumed the transition started when the value dropped below this threshold value.

**FIGURE 8:**
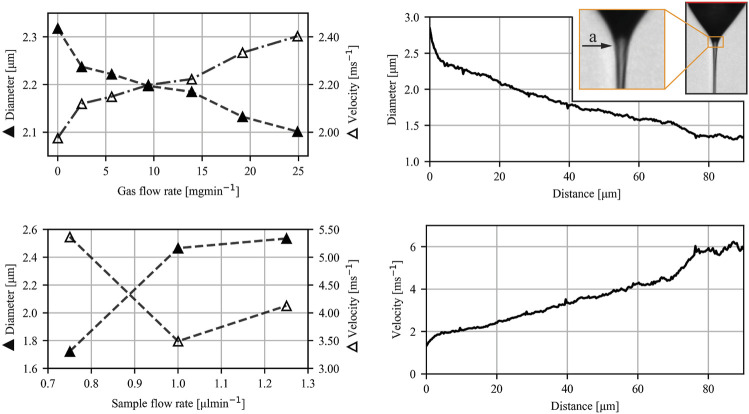
Diameter and velocity profiles for vacuum conditions. Figures on the left show the influence of GFR and SFR on jet diameter and velocity 50 μm downstream of the transition from the meniscus to the jet (denoted by an arrow (a) in the insert on the right). The figures on the right show a typical diameter of a jet (solid triangle) and its velocity profile (open triangle) in vacuum conditions. The dimensionless length is 0.405 for the lowest GFR values and 0.306 for the highest GFR values. EP = 6 kV for all cases.

Figure 8 shows the typical velocity and diameter profiles together with the influence of the gas and sample flow rates on the jet diameter and velocity in a vacuum. All figures display cases for which EP = 6 kV. The following parameters were used for the analysis of the velocity and diameter profiles: GFR = 19.2 mg/min, SFR = 0.5 μl/min. The influence of the gas flow rate on the jet was measured at constant SFR = 0.5 μl/min, and the influence of the sample flow rate on the jet was measured at constant GFR = 17.0 mg/min.

**FIGURE 9:**
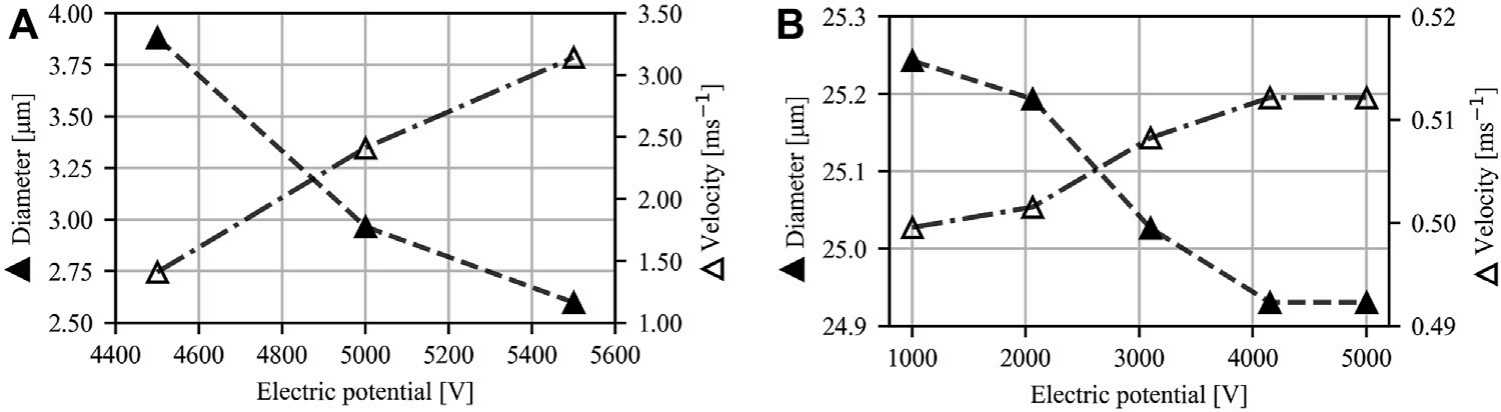
Diameter (solid triangle) and velocity profiles (open triangle) for vacuum conditions. Figures show the influence of EP on jet diameter and velocity 30 μm downstream of the meniscus to jet transition **(A)** and nozzle tip **(B)**. Parameters for **(A)**: GFR = 1.7 mg/min, SFR = 1 μl/min, *L* = 0.36 – 0.46 (high EP and low EP). Parameters for **(B)**: GFR = 30 mg/min, SFR = 15 μl/min, *L* = 0.75.

The effect of the applied voltage was investigated in a vacuum at constant gas and sample flow rates. Two instances of different sample flow rates are displayed in Figure 9. In the first example (Figure 9A) the sample flow rate was set to 1 μl/min, and the velocity was found to increase by more than twofold when the voltage was increased by 1 kV from an initial value of 4.5 kV. In the second example (Figure 9B) the sample flow rate was set to 15 μl/min, and the observed velocity increase was negligible over an extensive range of voltages (4 kV). At a lower sample flow rate, where jetting shown in Figures 4C, D occurs, the applied voltage plays more significant role on the jet’s velocity than at higher sample flow rate, where jetting shown in Figure 4B occurs.

### 3.4 Bridging regime

**FIGURE 10:**
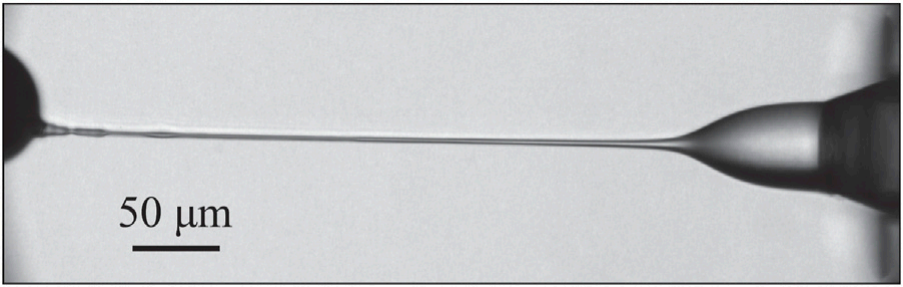
Bridging regime. GFR = 0 mg/min, SFR = 2 μl/min, EP = 6 kV, L = 1.

As mentioned above, at high sample flow and zero to low gas flow, a bridging regime occurs. Stable jetting can be achieved at specific flow rates, as shown in Figure 10. Similar to normal jetting modes, perturbations form and migrate downstream but do not trigger a breakup. The meniscus may detach and migrate downstream at a higher than stable sample flow rate, impacting the electrode. As mentioned earlier, the pulsing of the Taylor cone is often observed due to a small or absent stabilizing effect of the focusing gas.

Any parametrical combination of the EP-SFR-GFR values exhibiting pulsation or detachment under steady boundary conditions would correspond to a globally unstable behavior of the observed system. A detailed determination of the globally stable parametrical region would be a cumbersome task beyond the scope of the present work.

### 3.5 Observed parametric space

#### 3.5.1 Atmospheric pressure

**FIGURE 11:**
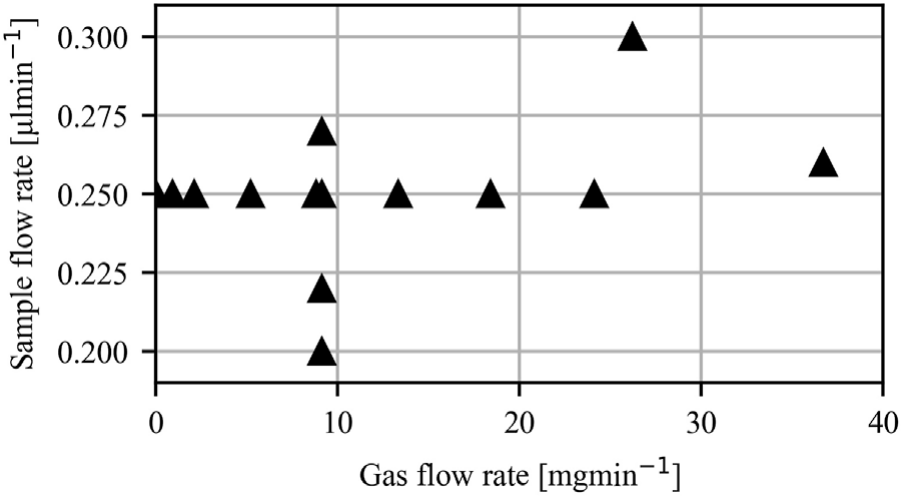
Tested parameter space where stable jetting was observed. EP = 7 kV for all cases.

Due to the low gas stabilizing and flow-focusing effect in an atmosphere environment, a stable jet was only obtained for EP values higher than 6.5 kV (Figure 11). The largest EP that was tested was 8 kV. GFR and SFR values were selected to showcase their effect on jet parameters and are, therefore, sparse. To ensure a stable jet over a range of GFR and SFR values, the EP was set to 7 kV in all recorded results. The lowest SFR, which still produced a stable jet, was around 0.2 μl/min, under which the Taylor cone and the jet chaotically formed and dissolved. The observed upper limit was around 0.3 μl/min, above which the Taylor cone underwent the pulsing. This narrow range of SFR could be attributed to the thin and short nature of the jets, which are unable to transport large mass flows. Stable operating modes were achieved with zero GFR and up to 40 mg/min, which was the highest tested GFR.

#### 3.5.2 Vacuum pressure

When operated in a vacuum, the higher stability of jets allowed us to explore a larger parametric space. Jetting modes 4c and 4b (Figure 4) were obtained for EP in the range of 4.5 kV–7 kV. Measurements in that range were taken in 500 V intervals. The region with the highest jet stability was observed at around 6-7 kV. SFR and GFR for modes 4c and 4b ranged between 0.5 and 3 μl/min and from 0 to 32 mg/min. Measurements for varying GFR were taken for a SFR in the range from 0.5 to 1 μl/min, where jetting was most stable. The same reasoning was used for measurements of varying SFR, where GFR ranged from 15 to 20 mg/min.

The measurements where the jetting mode 4b (Figure 4) occurs were taken at substantially higher SFR than for the modes observed in Figures 4C, D. The lowest SFR where regime 4b was observed is around 12 μl/min. The highest tested SFR was 30 μl/min. High gas flow rates were needed to produce a stable jet in mode 4b. The lowest observed was 30 mg/min. The highest tested GFR was 40.1 mg/min. EP plays no role on jet stability in mode 4b. A range of 0–8 kV was used for tests throughout the SFR range.

## 4 Discussion

An experimental study of liquid micro-jets produced with a GDVN under the influence of an electric field was carried out. The nozzle was designed to ensure direct imaging of the Taylor cone and its response to alternated flow rates and applied voltages. Additional work in nozzle design is needed to fully exploit the presence of an additional electrical force for producing highly accelerated micro-jets. The protruding capillary (as seen in Figures 1, 2) limits the transfer of kinetic energy from the expanding gas to the jet, limiting the effect of the flow focusing by the gas. Nevertheless, the presence of the applied voltage shows promising results regarding the additional jet acceleration and widening of the stable operating parametric space. While in conventional flow focusing techniques, the velocity of the jet reaches a plateau after the gas is fully expanded, no such plateau is reached in the presence of the applied voltage. It can be seen from Figures 7, 8 that the velocity of the jet increases linearly with distance. The positive effect of the voltage on the acceleration of the jet is also clearly presented in Figure 9A, albeit with a significant impact on the sample flow rate (Figure 9B). A further study of GDVN geometry modification to maximize the transfer of kinetic energy from the gas to the jet and, at the same time, preserve EP acceleration is needed. The improved GDVN that would provide the primary acceleration mechanism *via* the expansion of the gas could potentially use lower EP and therefore contribute to higher safety margins regarding the use of electric potential.

Another consideration regarding the design of the experiment for future work concerns the electrode. In the current work, a solid conically shaped electrode was used. While the positioning of such an electrode is straightforward, the problem is the deposition of the sample liquid on its surface. The rapid gas expansion in a vacuum causes the electrode to cool to subzero temperatures. The deposited sample film freezes and forms ice crystals that can grow up to the nozzle and clog the nozzle capillary. Therefore, in the present study, low sample and gas flow rates were used to delay the onset of ice deposition.

Further studies with more viscous sample liquids, such as octanol, are planned. As discussed previously, the molecular dissociation of the sample liquid is not negligible, so other, more stable liquids should be investigated. In addition, the excess of ionic species present due to a specific voltage polarity could play a significant role in the breakup length of the jet, motivating further research into this topic.

A high temporal resolution is required (Rubio et al., 2021) to reveal the exact breakup mechanisms of the jet. Based on the achieved velocities, the required resolution can be estimated somewhere between 10k and 100k FPS (10 µs–100 µs frame period). Concerning the breakup, a closer look at the secondary liquid ligaments issuing from the primary charged jet would also be of interest. In this regard, a numerical model is under development, based on our previous modelling experience (Zahoor et al., 2018a; Zahoor et al., 2018b; Zahoor et al., 2018c; Zahoor et al., 2020; Zahoor et al., 2021a; Zahoor et al., 2021b; Rubio et al., 2021; Šarler et al., 2021).

## Data Availability

The raw data supporting the conclusion of this article will be made available by the authors, without undue reservation.
